# Clinical Significance of EML4-ALK Fusion Gene and Association with EGFR and KRAS Gene Mutations in 208 Chinese Patients with Non-Small Cell Lung Cancer

**DOI:** 10.1371/journal.pone.0052093

**Published:** 2013-01-14

**Authors:** Ying Li, Yongwen Li, Tong Yang, Sen Wei, Jing Wang, Min Wang, Yuli Wang, Qinghua Zhou, Hongyu Liu, Jun Chen

**Affiliations:** Tianjin Key Laboratory of Lung Cancer Metastasis and Tumor Microenvironment, Tianjin Lung Cancer Institute, Tianjin Medical University General Hospital, Heping District, Tianjin, China; University Magna Graecia, Italy

## Abstract

The EML4-ALK fusion gene has been recently identified in a small subset of non-small cell lung cancer (NSCLC) patients who respond positively to ALK inhibitors. The characteristics of the EML4-ALK fusion gene in Chinese patients with NSCLC are poorly understood. Here, we report on the prevalence of EML4-ALK, EGFR status and KRAS mutations in 208 Chinese patients with NSCLC. EGFR mutations were found in 24.5% (51/208) of patients. In concordance with previous reports, these mutations were identified at high frequencies in females (47.5% vs 15.0% in males; *P*<0.05); never-smokers (42.3% vs 13.9% in smokers; *P*<0.05), and adenocarcinoma patients (44.2% vs 8.0% in non-adenocarcinoma patients; *P*<0.05). There were only 2.88% (6/208) patients with KRAS mutations in our study group. We identified 7 patients who harbored the EML4-ALK fusion gene (3.37%, 7/208), including 4 cases with variant 3 (57.1%), 2 with variant 1, and 1 with variant 2. All positive cases corresponded to female patients (11.5%, 7/61). Six of the positive cases were non-smokers (7.69%, 6/78). The incidence of EML4-ALK translocation in female, non-smoking adenocarcinoma patients was as high as 15.2% (5/33). No EGFR/KRAS mutations were detected among the EML4-ALK positive patients. Pathological analysis showed no difference between solid signet-ring cell pattern (4/7) and mucinous cribriform pattern (3/7) in ALK-positive patients. Immunostaining showed intratumor heterogeneity of ALK rearrangement in primary carcinomas and 50% (3/6) of metastatic tumors with ALK-negative staining. Meta-analysis demonstrated that EML4-ALK translocation occurred in 4.84% (125/2580) of unselected patients with NSCLC, and was also predominant in non-smoking patients with adenocarcinoma. Taken together, EML4-ALK translocations were infrequent in the entire NSCLC patient population, but were frequent in the NSCLC subgroup of female, non-smoker, adenocarcinoma patients. There was intratumor heterogeneity of ALK rearrangement in primary carcinomas and at metastatic sites.

## Introduction

Lung cancer is a leading cause of cancer-related deaths, where it is associated with a 5-year worldwide survival rate of less than 15% [1], [2]. Non-small-cell lung cancer (NSCLC) accounts for approximately 80% of lung cancers [3]. Cisplatin-based chemotherapy has produced a significant survival benefit over best supportive care in patients with advanced NSCLC; however, the outcomes for NSCLC patients are still considered unsatisfactory [3], [4]. Traditional chemotherapy and radiotherapy are associated with significant side effects in all patients with NSCLC, owing to their lack of specificity. The development and application of drugs that target specific molecules expressed on lung cancer cells has garnered increased attention, and remarkable successes have been reported in several NSCLC patient study groups [5], [6], [7], such as a subset of patients with NSCLC testing positive for activating mutations in the epidermal growth factor receptor (EGFR) gene. These cancers exhibit sensitivities to EGFR tyrosine kinase inhibitors (TKIs), such as gefitinib or erlotinib, supporting the use of these drugs for effective treatment of NSCLC [5], [8]. Among patients carrying the EGFR activating mutation, who have previously untreated, advanced disease, gefitinib has been demonstrated to be superior to cytotoxic chemotherapy [4], [9], [10]. The utility of EGFR TKIs in these patients highlights the importance of identifying genotype-specific patient subsets to guide the selection of targeted therapies.

Recently, a fusion protein between the N-terminal portion of the echinoderm microtubule-associated protein-like 4 (EML4) protein and the intracellular signaling portion of the anaplastic lymphoma kinase (ALK) tyrosine kinase receptor has been identified in a small subset of NSCLC patients [11]. Patients harboring the EML4-ALK fusion protein demonstrate unique clinical pathological and physiological characteristics [12], [13]. Several reports have identified this fusion protein predominantly in young female non-smokers with adenocarcinoma [11], [14], whereas other reports have described this fusion protein in different populations [15], [16]. The small overall number of positive instances identified per study may explain these discrepancies. The characteristics of EML4-ALK in lung cancers remain to be elucidated, particularly in Chinese patients with NSCLC. Is the prevalence of EML4-ALK mutations, just like the EGFR mutation, linked to one specific ethnicity?

The EGFR/KRAS mutations and the EML4-ALK translocation are called driver mutations because they are responsible for both the initiation and maintenance of lung cancer. Adenocarcinomas exhibit distinct genomic changes that can be classified into clinically relevant molecular subsets according to KRAS, EGFR, and EML4-ALK status. Specific mutations in these genes induce differential tumor sensitivities to targeted therapeutic agents. For instance, tumors carrying dominant somatic mutations in EGFR exons are typically sensitive to EGFR-TKIs. Tumors harboring somatic mutations in KRAS, which encodes a GTPase downstream of EGFR, display greater resistance to these drugs. Therefore, it is necessary to understand the correlations between EML4-ALK and EGFR/KRAS mutations in lung cancer patients.

Here, we report on the large-scale clinical and pathological characteristics of this fusion in 208 Chinese NSCLC patients, and we correlate it with the presence of EGFR and KRAS mutations in the same patients. We also present a meta-analysis of the EML4-ALK fusion gene.

## Results

### Clinical characteristics of the EML4-ALK fusion gene in Chinese NSCLC patients

**Figure 1:**
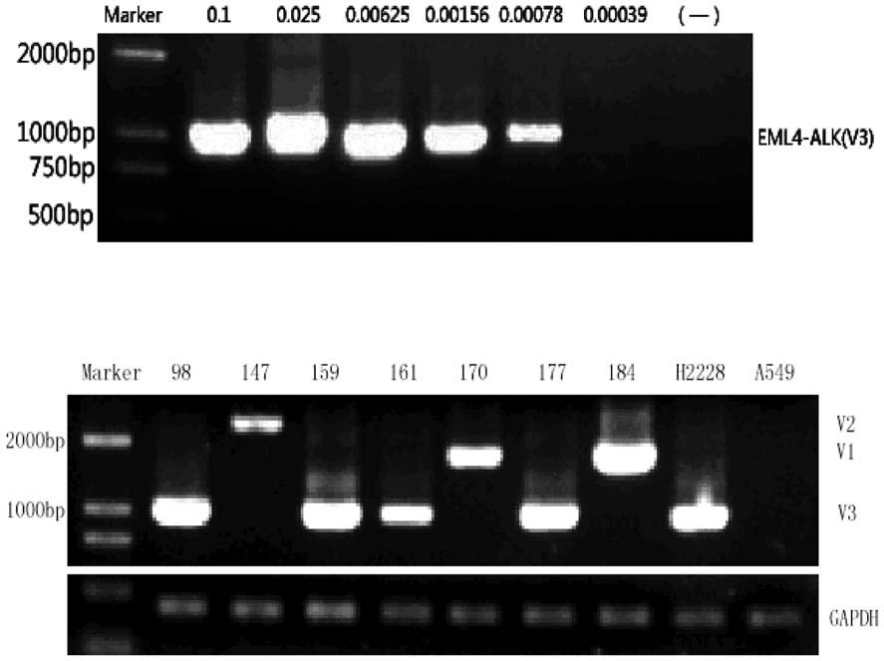
Detection of EML4-ALK translocation by nested RT-PCR. Nested RT-PCR was performed using serially diluted cDNA from H2228 cells. As little as 7.8×10^−4^ ng cDNA could be used for consistent detection of the variant 3 fusion transcript with this protocol (Upper panel). Representative gel electrophoresis results for the nested RT-PCR (lower panel). Lane marker: 200-bp ladder; positive control: H2228; negative control: A549. Other lanes correspond to samples exhibiting the EML4-ALK translocation, labeled by case number.

In 2007, Martelli et al. evaluated patients who expressed EML4-ALK transcripts, using fluorescence *in situ* hybridization, and found that only approximately 1.8% of cells harbored the EML4-ALK gene fusion [17]. These researchers were unable to detect the EML4-ALK protein by immunohistochemistry, Western blotting, or immunoprecipitation. RT-PCR is the most sensitive and most popular detection method currently available. We performed nested RT-PCR to amplify the fusion region of the translocation. We assessed the sensitivity of our nested RT-PCR protocol by amplifying serially diluted cDNA from H2228 cells. The nested RT-PCR detected the EML4-ALK fusion gene at a lower limit of 7.8×10^−4^ ng input cDNA (Figure 1A).

**Figure 2:**
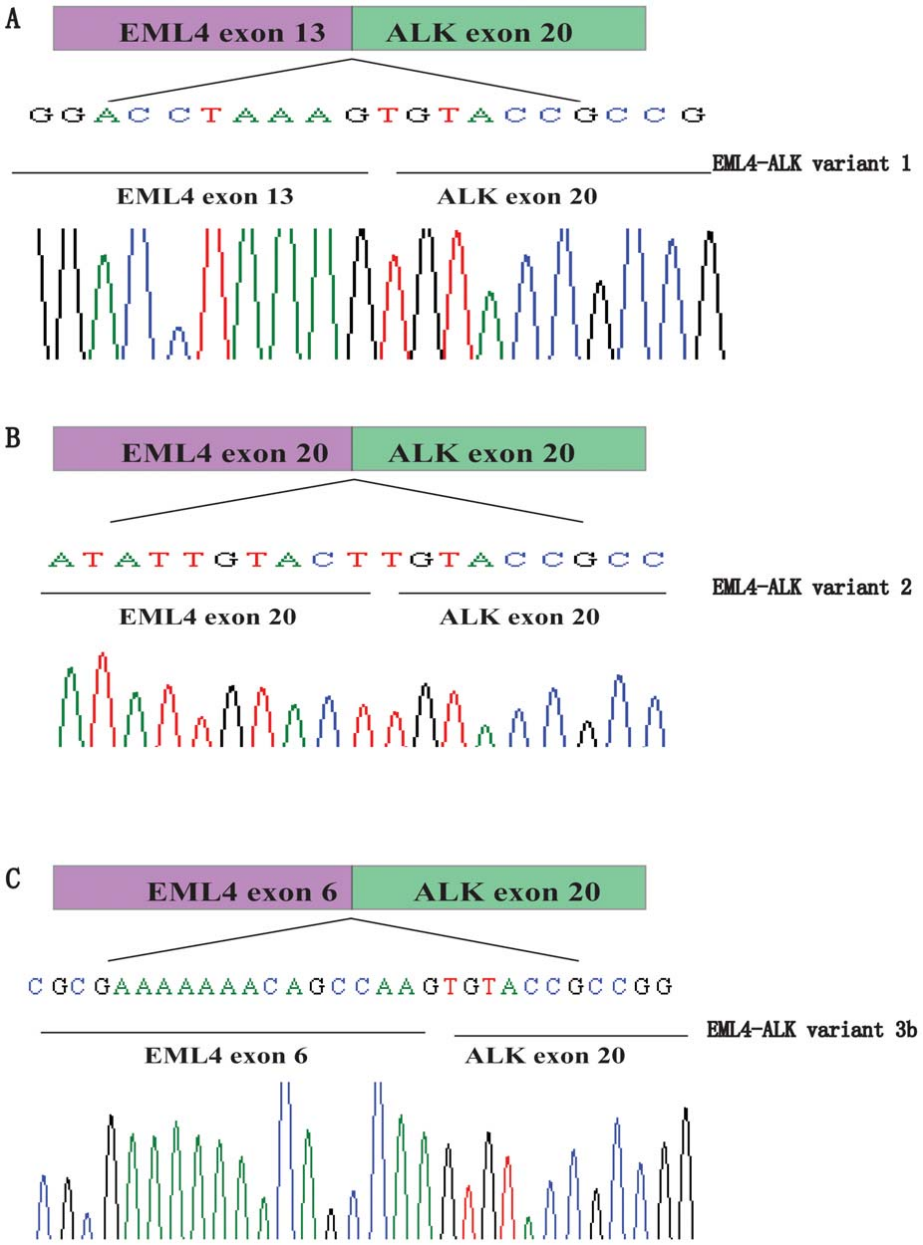
Schematic representation of fusion junctions and flanking sequences of the EML4-ALK fusion gene variants. (A) variant 1, (B) variant 2, (C) variant 3.

There were 208 NSCLC patients in our study and their clinical and pathological characteristics are detailed in Table 1. The results of nested RT-PCR amplifications of the EML4-ALK fusion gene are shown in Figure 1B. We identified 7 patients who harbored the EML4-ALK fusion gene (3.37%, 7/208), which was confirmed by DNA sequencing (Figure 2). Of these 7 patients, 2 cases displayed the EML4-ALK variant 1 (28.6%, 2/7), 1 case exhibited variant 2 (14.3%, 1/7) and 4 cases carried variant 3 (57.1%, 4/7). Therefore, variant 3 may be the predominant variant among Chinese NSCLC patients with more than half of the EML4-ALK translocations exhibiting fusions between exon 6 of EML4 and exon 20 of ALK.

**Table 1:** NSCLC patient characteristics (N = 208).

Characteristic		N (%)
Gender	Male	147 (70.8%)
	Female	61 (29.2%)
Age	>60	120 (57.7%)
	≤60	88 (42.3%)
Histology	AD	95 (46.7%)
	SCC	96 (46.2%)
	ASC	7 (3.4%)
	Others	10 (4.8%)
Smoking history	Non-smoker	78 (37.5%)
	Smoker	130 (62.5%)
Differentiation	Well	32 (15.4%)
	Moderate	70 (33.7%)
	Poor	106 (51.0%)
Metastasis	None	75 (36.1%)
	Yes	133 (63.9%)
Stage	I	49 (23.6%)
	II	43 (20.7%)
	III	106 (51.0%)
	IV	10 (4.8%)

The clinical characteristics of these 7 patients are described in Table 2. Six cases involved adenocarcinoma and one case involved adenosquamous carcinoma. Tests for other types of carcinoma were negative (0/106). As shown in Table 3, the prevalence of the EML4-ALK fusion gene was 6.32% (6/95) among adenocarcinoma cases and 14.2% (1/7) among adenosquamous carcinoma cases. All positive cases corresponded to female patients, resulting in a positive rate among females of 11.5% (7/61; *p* = 0.000). Six of the positive cases were non-smokers (7.69%, 6/78). The frequency of the EML4-ALK translocation among female non-smoking adenocarcinoma patients was 15.2% (5/33). The median age of EML4-ALK-positive cases (55±10.38) was much lower than that of EML4-ALK-negative patients (61.78±8.71; *p* = 0.045). No significant associations were detected between the gene fusion and stage or metastatic status.

**Table 2:** Clinical features associated with EML4-ALK fusion gene-positive patients.

*Case number*	*Age*	*Gender*	*Smoking status*	*Histology (H&E)*	*Differentiation*	*P-TNM*	*Metastasis status*	*EML4-ALK variant* *	*ALK-IHC/IF at primary sites*	*ALK-IHC/IF at metastatic sites*	*EGFR/KRAS mutation*	Survival
1-147	66	F	Non	AD	Poor	T2aN2M0	M	2	+	−	-/-	Y
2-159	52	F	Non	Mixed-AD	Moderate	T1N2M0	M	3	+	+	-/-	N
3-161	56	F	Non	Mixed -AD	Poor	T1N2M0	M	3	+	−	-/-	Y
4-170	46	F	Non	Ad+SCC	Poor	T2bN2M0	M	1	+	−	-/-	N
5-177	52	F	Non	Mixed -AD	Poor	T2aN1M0	M	3	+	+	-/-	Y
6-184	42	F	Y	Mixed -AD	Poor	T2aN1M1	M	1	+	+	-/-	Y
7-98	71	F	Non	Mucinous BAC	Well	T2aN0M0	N	3	+		-/-	Y

* Variant 1: EML4 E13-ALK E20; Variant 2: EML4 E20-ALK E20; Variant 3: EML4 E6-ALK E20. +: positive staining; −: negative staining.

**Table 3:** Clinical characteristics of genotype-specific subsets of NSCLC patients.

Characteristic	Genotype								
	EML4-ALK(n = 7)			EGFR (n = 51)			KRAS (n = 6)		
	No.	%	p	No.	%	p	No.	%	p
Age (years)			0.042			0.064	63.5	2.9	0.992
Median	55	3.35		61.78	24.4		53-72		
Range	42-71			33-83					
Sex			0.000			0.000			0.49
Male	0	0		22	43.1		5	83.3	
Female	7	100		29	56.9		1	16.7	
Pathology			0.033			0.000			0.289
Adeno	6	85.7		42	82.4		4	66.7	
Adenosquamous	1	14.3		3	5.9		0	0	
Squamous	0	0		5	9.8		1	16.7	
Large cell	0	0		1	2.0		0	0	
Others	0	0		0	0		1	16.7	
Smoking status			0.007			0.000			0.286
Smoker	1	14.3		19	37.3		5	83.3	
Never smoker	6	85.7		32	62.7		1	16.7	
Stage			0.614			0.064			0.293
I	1	14.3		10	19.6		0	0	
II	1	14.3		9	17.6		2	33.3	
III	4	57.1		26	51.0		3	50.0	
IV	1	14.3		6	11.8		1	16.7	
Metastasis			0.223			0.153			0.317
Yes	6	85.7		37	72.5		5	83.3	
No	1	14.3		14	27.5		1	16.7	

### Histological analysis of EML4-ALK-positive lung cancer

**Figure 3:**
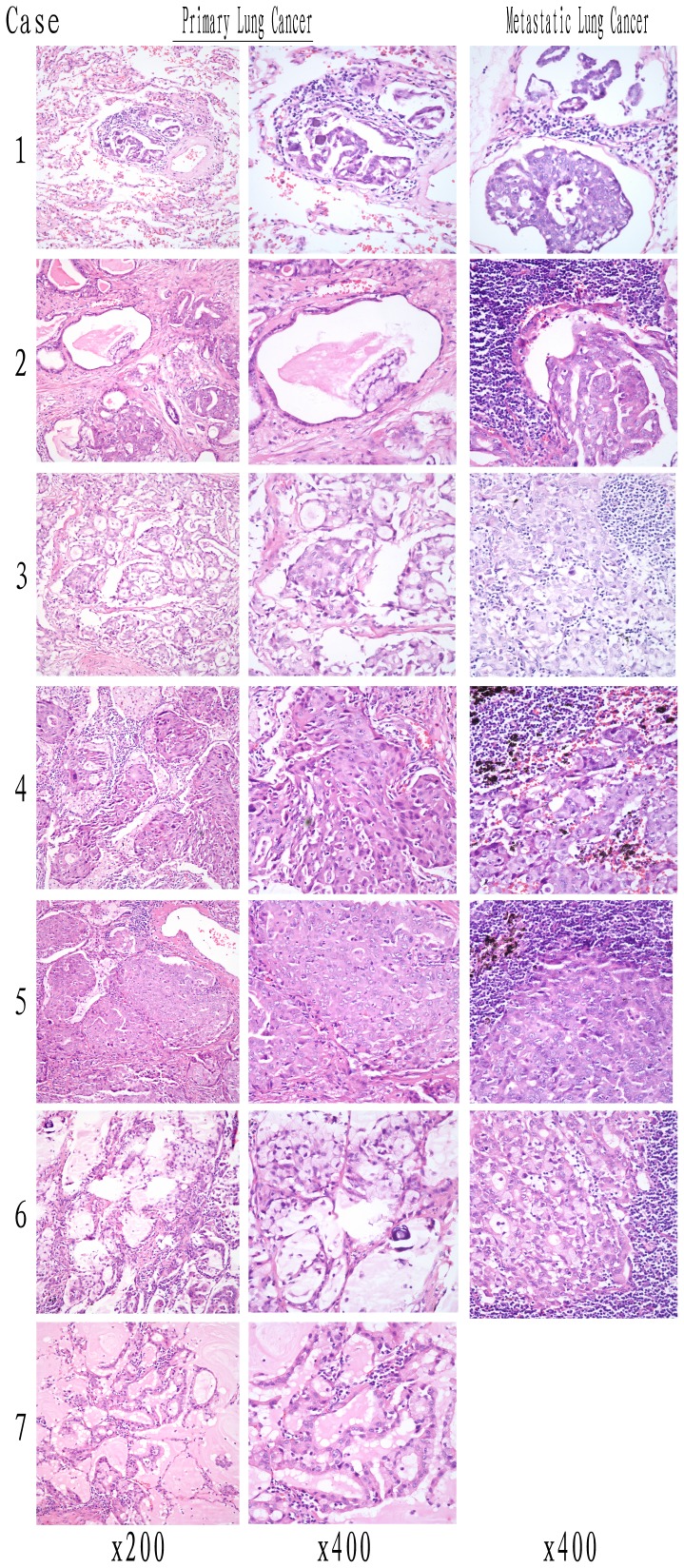
The pathological analysis of EML4-ALK-rearranged lung cancer patients by H&E staining. The primary tumors of Cases 1, 3, 4 and 5 showed the solid signet-ring cell pattern composed of solid growth with papillary (or micropapillary) or a signet-ring cell component, and other primary sites of Cases 2, 6 and 7 showed the mucinous cribriform pattern consisting of abundant extracellular mucus and cribriform structures. In the lymph node metastatic sites, solid signet-ring cell pattern was identified in Cases 1–5, and mucinous cribriform pattern was only seen in Case 6.

As shown in Figure 3, all 7 of the ALK-positive tumors at least harbored an adenocarcinoma component. Only one case (Case 4) had focal squamous differentiation (accounting for 20% of the tumor volume). The adenocarcinoma component showed a varied histomorphology, and the predominant growth pattern was papillary (or micropapillary) in 1 case (Case 1), acinar in 3 cases (Cases 2, 6 and 7), and solid in 3 cases (Cases 3, 4 and 5). However, most cases (5/7) actually had a mixed adenocarcinoma component including papillary or micropapillary, signet-ring cells, and mucous cells. On the basis of the previously reported pathological pattern for ALK-positive carcinomas [18], 57.14% (4/7, Cases 1, 3, 4 and 5) of ALK-positive cases showed the solid signet-ring cell pattern and 42.86% (3/7, Cases 2, 6 and 7) of cases showed the mucinous cribriform pattern. A psammoma body was also identified in Case 6, which was a mixed adenocarcinoma with signet-ring cells, mucous cells and micropapillary component. Interestingly, we found a purely noninvasive mucinous BAC (bronchioloalveolar carcinoma) in Case 7, in which goblet cells showed a lepidic growth pattern. Tissues from metastatic lymph nodes were available in 6 of the ALK-positive cases. Papillary or micropapillary, signet-ring cells with extracellular mucus, and cribriform structure were identified in 2 (Cases 1 and 2), 1 (Case 6), and 3 (Cases 3, 4 and 5) cases, respectively. Five cases showed the solid signet-ring cell pattern, and 1 case (Case 6) showed the mucinous cribriform pattern.

In addition, a 3-tier grading system of lung adenocarcinomas based on histological pattern was reported by Sica et al.[19], including Grade I: a pattern with low grade (BAC), Grade II: pattern with intermediate grade (acinar and papillary); and Grade III: pattern with high grade (solid and micropapillary). Using this grading system, only Case 7 belonged to Grade I, and all the rest belonged to Grade III since most of them were mixed adenocarcinomas with lymph node metastasis.

**Figure 4:**
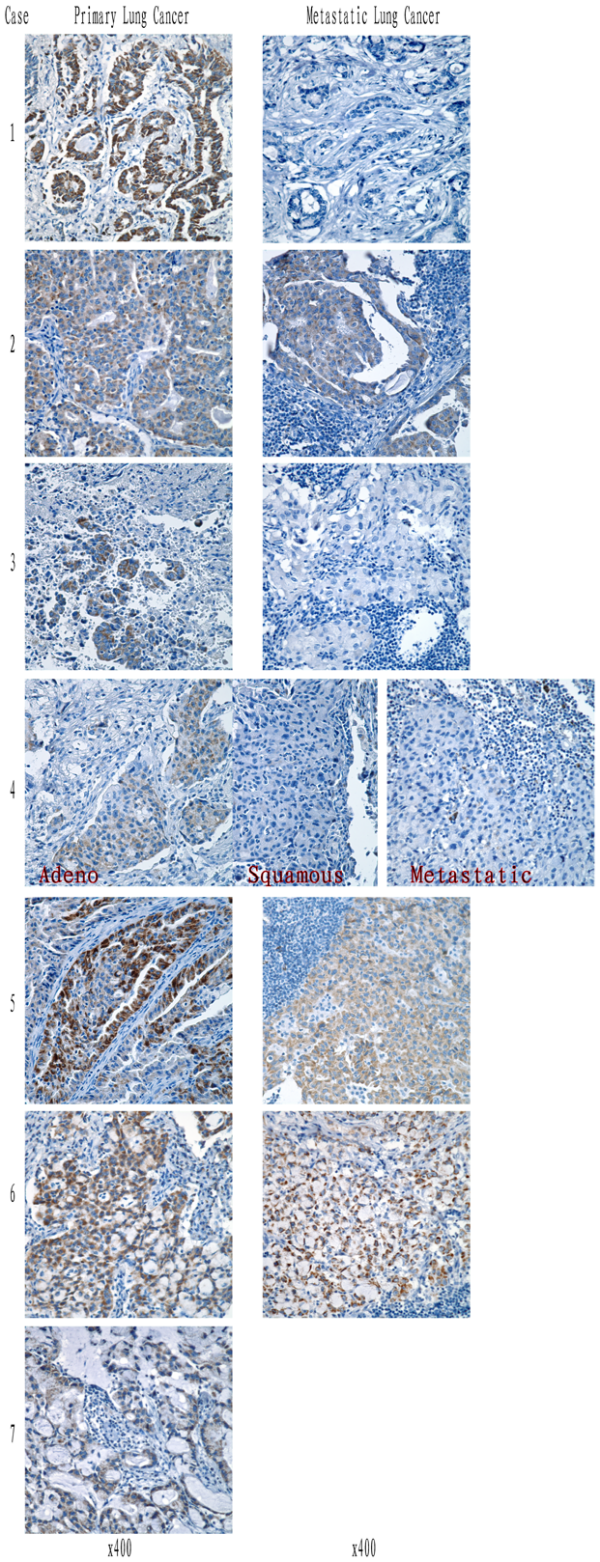
The pathological analysis of EML4-ALK-rearranged lung cancer patients by ALK immunohistochemistry staining. ALK-positive immunostaining was shown in primary tumors of all 7 ALK-rearranged lung cancer patients with diffuse staining in the cytoplasm. ALK-positive staining was not apparent in each primary tumor cell, especially in Case 4, which is an adenosquamous carcinoma with ALK positive staining only in the adenocarcinoma part, not in the squamous carcinoma component (indicated in red text: Adeno and Squamous). For the lymph node metastatic sites, Cases 2, 5 and 6 showed ALK-positive staining and Cases 1, 3 and 4 showed ALK-negative staining.

Next, we performed ALK immunohistochemistry staining (ALK-IHC) of primary and metastatic tumors of all ALK-positive cases. As shown in Figure 4, ALK-IHC was positive in all primary tumors, and ALK protein expression was located in the cytoplasm. Moreover, the ALK-positive staining was not seen in each primary tumor cell. It was very apparent in Case 4 with the adenosquamous carcinoma, where ALK immunostaining occurred in the adenocarcinoma component but not in the squamous carcinoma component. This result indicated a phenomenon of intratumor heterogeneity of ALK rearrangement in the primary tumor sites. Furthermore, there were only 3 cases (Cases 2, 5 and 6) with ALK-positive staining in the metastatic tumors. The other 50% of the metastatic tumors were completely ALK-negative staining, including Cases 1, 3 and 4. For Case 4, even though the metastatic tumors comprised most of the adenocarcinoma component, ALK immunostaining was still completely negative.

**Figure 5:**
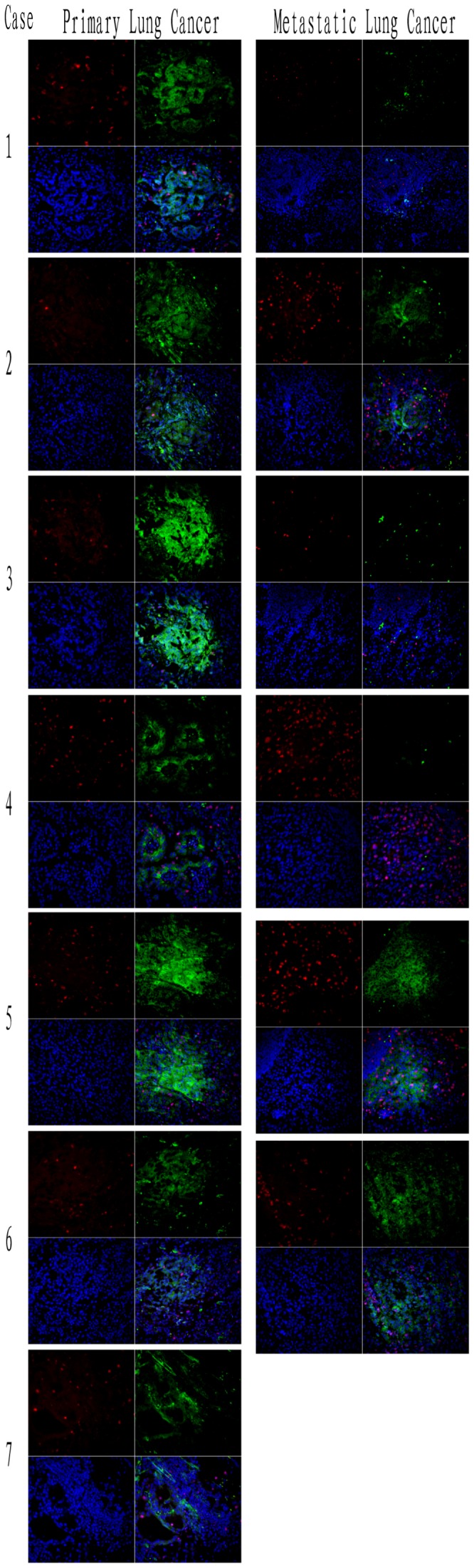
The pathological analysis of EML4-ALK-rearranged lung cancer patients by ALK immunofluorescence staining. ALK-positive immunostaining was shown in primary tumor of all 7 ALK-rearranged lung cancer patients. For the lymph node metastatic sites, Cases 2, 5 and 6 showed ALK-positive staining and Cases 1, 3 and 4 showed ALK-negative staining. These results were similar to those for ALK immunohistochemistry. ALK: green; Ki-67: red; DAPI: blue.

Finally, ALK immunofluorescence staining (ALK-IF) was also employed in ALK-IHC of the primary and metastatic tumors in all ALK-positive cases, which further confirmed the ALK-IHC findings. As shown in Figure 5, ALK-IF was also positive in all primary tumors, with the ALK protein localizing in the cytoplasm. Similar results were obtained with ALK-IHC, where 50% of the metastatic sites showed complete ALK-negative staining in Cases 1, 3 and 4. We also performed RT-PCR for the EML4-ALK gene with cDNA from all metastatic tumors and obtained the same results, that is, negative expression of the EML4-ALK gene in Cases 1, 3 and 4 (data not shown). All these data confirmed the intratumor heterogeneity of ALK rearrangement in the primary lung carcinomas.

### EGFR and KRAS mutations and clinical characteristics of patients

Of the 208 patients studied, we identified 51 cases of EGFR mutations in exons 18, 19, 20, or 21 (24.5%, 51/208; Table S1). Mutations in exon 18 of EGFR were identified in 3 cases; an exon 19 deletion was identified in 21 cases, and mutations in exons 20 and 21 mutations were detected in 6 and 26 cases, respectively. Five cases exhibited double mutations in those EGFR exons. Among exons 18–21, EGFR mutations occurred predominantly in exons 19 and 21 (90.19%, 46/51). In concordance with previous reports, the EGFR mutation rate in adenocarcinoma patients (44.2%, 42/95) was much higher than that in non-adenocarcinoma patients (8.0%, 9/113; *P* = 0.000). In addition, the EGFR mutation rate was significantly lower in male patients (15.0%, 22/147) than in female patients (47.5%, 29/61; *P* = 0.000). Among non-smokers, the EGFR mutation rate was 42.3% (33/78), much higher than that among smokers (13.9%, 18/130; *P* = 0.000). No associations were identified between EGFR gene mutations and age, clinical stage, tumor size, or metastasis status of the patients (Table 3).

Among 208 patients, 6 cases carried KRAS mutations (2.88%, 6/208). No associations were detected between the KRAS mutations and age, clinical stage, gender, pathology, smoking status, tumor size, or metastasis status of the patients (Table 3). No EGFR/KRAS mutations were detected among EML4-ALK-positive patients in our study.

### Survival analysis

As of March 2011, 181 patients had complete prognosis data, including 49 deaths and 132 cases of survival. Median follow-up for the entire series was 591 days (range from 0 days to 1329 days). We conducted a survival analysis of these 181 patients with respect to the presence of EML4-ALK fusion genes and EGFR/KRAS mutations. We identified 45 patients with EGFR mutations, for whom median survival time was 19.3 months (578 days; 124–1281). Of the 136 patients with normal EGFR, the median survival time was 19.8 months (594.5 days; 0–1329). The difference in median overall survival times between these groups was not statistically significant (*P* = 0.467). We identified 4 patients with KRAS mutations for whom median survival time was 16.3 months (489 days; 233–818). Of the 177 patients with normal KRAS, the median survival time was 19.7 months (592 days; 0–1329). Among the 181 cases, 7 patients carried the EML4-ALK fusion gene translocation. The median follow-up for these patients was 16.8 months (504 days; 360–661), which was not significantly different compared to the EML4-ALK negative group with 33.4 months (594.5 days; 0–1329) (*P* = 0.655).

### Meta-analysis of clinical characteristics associated with EML4-ALK translocation

**Figure 6:**
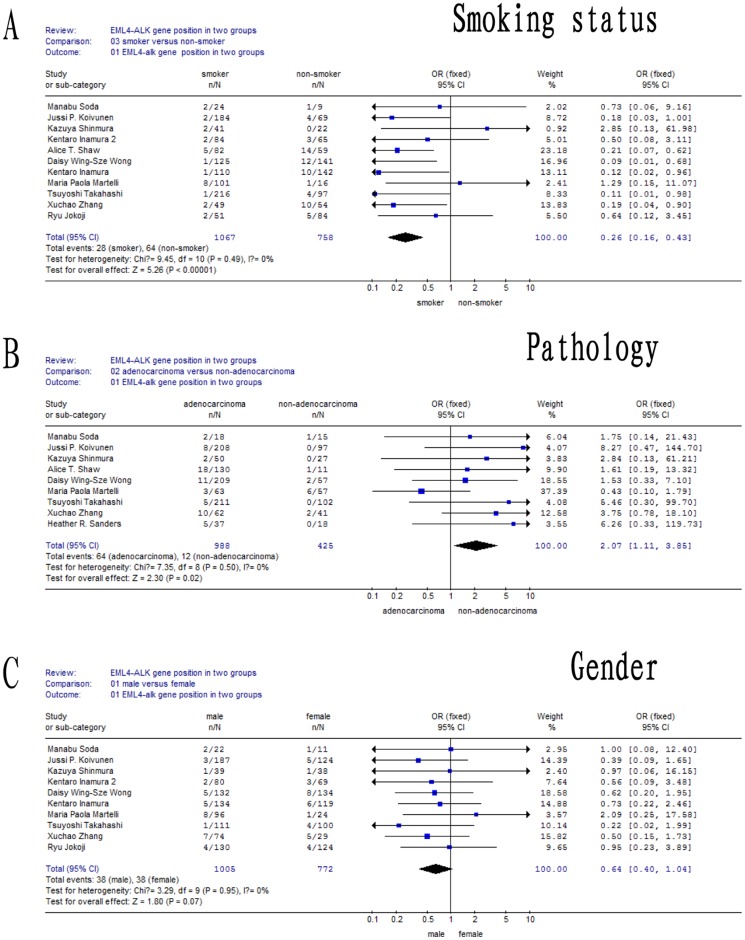
Meta-analysis of data for EML4-ALK. (A) Meta-analysis using a random effects model demonstrated that smoking status was significantly correlated with EML4-ALK translocation; (B) Meta-analysis of data using a fixed effects model indicated that adenocarcinoma (AD) significantly correlated with the EML4-ALK translocation. The translocation was significantly more frequent in the AD group than in the non-AD group; (C) Meta-analysis using a fixed effects model suggested that gender was not significantly correlated with EML4-ALK translocation frequency.

As shown in Table 4, systematic analysis of the literature revealed 14 publications analyzing the frequency and clinical characteristics associated with EML4-ALK translocation, including 2580 NSCLC patients. Of these, 125 patients (4.84%, 125/2580) harbored the EML4-ALK rearrangement. The meta-analysis demonstrated that EML4-ALK translocation was predominant in female patients with adenocarcinoma. Eleven studies (1825 cases) examined a correlation between smoking status and the EML4-ALK translocation. We detected a significant bias between the smoking and non-smoking groups (*P* = 0.49), so data were analyzed using a random effects model. A significant difference was detected in EML4-ALK positivity status between smokers and non-smokers (OR [95% CI] = 0.26 [0.16, 0.43], Z = 5.26; *P<0.05*) (Figure 6A). These results indicated that smoking status was significantly associated with the EML4-ALK translocation. Nine reports (1413 cases) assessed the EML4-ALK translocation in adenocarcinoma and non-adenocarcinoma groups. We detected no significant bias between the two groups (*P* = 0.50), so a fixed effects model was used. Our results suggest that the EML4-ALK translocation frequency differed in the adenocarcinoma group compared to the non-adenocarcinoma group (OR [95% CI] = 2.07 [1.11, 3.84], Z = 2.30; *P<0.05*) (Figure 6B). Eleven studies (1777 cases) evaluated the EML4-ALK translocation in male and female groups. We found no significant bias between the two groups (*P* = 0.95) and analyzed the data using a fixed effects model. Our results indicated that there was no significant difference between the male and female groups. (OR (95%CI) = 0.64[0.40,1.04] Z = 1.80, *P*>0.05) (Figure 6C).

**Table 4:** Data extracted from 14 studies included in the meta-analysis.

*Author (N)*	*Frequency*	*Origin*	Method
Sanders et al.[27]	9.09% (5/55 )	Caucasian	RT-PCR
Jokoji et al. [28]	3.15% (8/254)	Asian	IHC
Zhang et al [14]	11.6% (12/103 )	Asian	RACE-coupled PCR sequencing
Takahashi et al.[12]	1.60% (5/313 )	Asian	RT-PCR
Lin et al [29]	11.3% (12/106 )	Caucasian	exon array and FISH
Shaw et al [15]	13.5% (19/141 )	Asian	FISH
Inamura et al [30]	3.03% (11/363 )	Asian	RT-PCR, FISH
Wong et al [16]	4.89% (13/266 )	Asian	RT-PCR
Martelli et al [22]	7.5% (9/120)	Caucasian	RT-PCR
Takeuchi et al [31]	4.35% (11/253 )	Asian	RT-PCR
Koivunen et al [32]	2.62% (8/305 )	Caucasian, Asian	RT-PCR
Shinmura et al [33]	2.60% (2/77 )	Asian	RT-PCR
Inamura et al [34]	3.36% (5/221 )	Asian	RT-PCR
Soda et al [11]	6.67% (5/75 )	Asian	RT-PCR

## Discussion

Lung cancer is a devastating disease associated with a 5-year survival rate of approximately 10–15%. Although new chemotherapeutic drugs have been developed during the past two decades, there is still an urgent need for more effective treatments. Molecularly targeted therapies present a new strategy that recently demonstrated the importance of sub-classifying patients according to tumor-specific biologic characteristics.

EGFR and KRAS are two relevant molecules in NSCLC, and EGFR is targeted by EGFR-TKIs. These drugs show great potential in the treatment of patients with advanced NSCLC. Numerous reports have confirmed that the efficacy of EGFR-TKIs correlates with EGFR exon mutations [5], [8], and that the incidence of EGFR mutations is higher among Asians, non-smokers, females, and patients with adenocarcinoma. Soda et al. (2007) suggested an additional potentially relevant oncogenic event in lung cancer: fusion of the EML4 gene (region corresponding to the N-terminal segment) with the ALK gene (region corresponding to the intracellular signaling portion) to create an EML4-ALK fusion gene. The EML4-ALK translocation represents a new subgroup of NSCLC patients who respond positively to ALK inhibitors [11], [20].

Our work confirms the incidence (24.5%) of EGFR mutations in an unselected Chinese population of patients with NSCLC. Among exons 18–21, EGFR mutations occur predominantly (90.19%) in exons 19 and 21. In concordance with previous reports, these mutations are identified at high frequencies in females, never-smokers, people of Asian ethnicity, and adenocarcinoma patients. Interestingly, the incidence (6/208, 2.88%) of KRAS mutations in our study group was very low compared with Western countries (12–25%) [21].

Our work also confirms the low incidence (7/208, 3.37%) of the EML4-ALK translocation among unselected NSCLC patients, which is consistent with the meta-analysis of clinical characteristics associated with the EML4-ALK translocation in the published literature (4.84%). This result indicates that the prevalence of EML4-ALK mutations in the Chinese population is similar to that previously published for populations of other ethnicities. The EML4-ALK translocation may not be linked to one specific ethnicity, unlike EGFR mutations in Asians. Furthermore, the analysis of EML4-ALK mutation variants revealed that variant 3 may be the predominant variant among Chinese NSCLC patients with more than half of the EML4-ALK translocations exhibiting fusions between exon 6 of EML4 and exon 20 of ALK. These results further suggest that the prevalence of EML4-ALK mutation may not vary by ethnicity but that a particular variant may be prevalent in one specific ethnic group.

Our results further indicate that the EML4-ALK translocation occurs predominantly in females, never-smokers, and adenocarcinoma patients. These results were different from the meta-analysis of gender, in which there was no significant difference between the male and female groups. In our study, all 7 ALK-positive cases were female, and the reason may be due to the much higher smoking rate in men than that in women in China. Further analysis proved that the incidence of EML4-ALK translocation in female, non-smoking, adenocarcinoma patients is as high as 15.2%. These characteristics are of great significance, because female, non-smoking, adenocarcinoma patients are typically selected to benefit from EGFR-TKI treatment, indicating that an EML4-ALK translocation screen should precede TKI treatment. The EGFR/KRAS mutations and the EML4-ALK translocation are called driver mutations because they are responsible for both the initiation and maintenance of lung cancer. Patients who harbor the EML4-ALK translocation are usually not responsive to EGFR-TKI treatment, but may be sensitive to ALK inhibitors [11], [20].

In our study, comparison of EML4-ALK fusion status with the presence of EGFR and KRAS mutations in the same cancer samples revealed that EML4-ALK fusions occurred in the absence of EGFR or KRAS mutations. Although previous reports have indicated that ALK fusion can occur concurrently with EGFR mutations (1/305) and KRAS mutations (1/120), these may be rare events [7], [22]. Further analysis indicated in the 32 cases of non-smoking, female, adenocarcinoma patients that the frequency of EGFR exon mutations and EML4-ALK translocation were 62.5%% (20/32) and 15.63% (5/32), respectively, which covers all 78.13% (25/32) of these patients. In addition, Sasaki et al. recently reported that 6% (3/50) of treatment-naïve NSCLC patients with ALK rearrangement had concurrent EGFR activating mutations and that the ALK rearrangement was inactive in these patients due to EGFR mutation [23]. Since most patients with EML4-ALK fusions do not exhibit EGFR mutations, a specific molecular subset of adenocarcinomas is characterized by EML4-ALK fusion [14]. Today, some specific molecular drugs for EML4-ALK fusion are used to treat NSCLC patients with EML4-ALK translocation. Therefore, a stepwise approach to test for gene mutations in lung adenocarcinoma is suggested: first for KRAS, second for EGFR, and then EML4-ALK translocation. If a tumor is positive for a KRAS mutation, no further molecular testing is required, and treatment recommendations will focus on chemotherapy because tumors harboring somatic mutations in KRAS, which encodes a GTPase downstream of EGFR, display greater resistance to these targeted drugs. If the tumor is negative, it will be tested for EGFR mutations and EML4-ALK translocation. A positive result for either EGFR mutation or EML4-ALK translocation will trigger a targeted treatment: an EGFR TKI or an ALK inhibitor.

For the pathological analysis, cribriform structure, presence of mucous cells, extracellular mucus, and lack of significant nuclear pleomorphism, could all be seen in the EML4-ALK rearranged cancer, which was similar to findings in a previous report [18]. “Solid signet-ring cell pattern” and “mucinous cribriform pattern” are two recognizable cytoarchitecures present at least focally in the majority of ALK-positive tumors [18]. With regard to these two patterns, we did not see a significant difference between solid signet-ring cell pattern and mucinous cribriform pattern in all ALK positive cases in our study (4/7 vs 3/7, respectively). Interestingly, our ALK immunostaining results indicated that not all tumor cells were ALK-positive staining at the primary sites, especially in adenosquamous carcinoma, in contrast to other studies reporting that the squamous carcinoma component stains positive [18], [24]. Even more surprisingly, we found that 50% of the metastatic tumors were completely negative for ALK immunostaining (IHC and IF). This phenomenon was also found in other studies with EGFR mutations, which showed 38.0% of the tumors (30 of 79) having an intratumor heterogeneity of *EGFR* mutations and 62.0% (49 of 79) being homogeneous, either with *EGFR* mutation or no mutation [25]. Our immunostaining also confirmed intratumor heterogeneity of ALK rearrangement in primary tumors, and to our knowledge, this is the first report on ALK rearrangement in lung cancer at metastatic sites versus primary sites, which showed molecular differences. These results indicate that gene expression in the metastatic tumor is not completely similar to that in the primary tumor. For the patients treated with the molecular target drugs, these results also point out that the new molecular detection is very necessary for these patients with new metastatic sites, especially for NSCLC patients with EML4-ALK translocations.

In summary, we confirm that EGFR exon mutations are frequent in patients with NSCLC, especially among females, non-smokers, and adenocarcinoma patients. EML4-ALK translocations are infrequent in the entire NSCLC patient population, but are frequent in the NSCLC patient subgroup of female, non-smoking, adenocarcinoma patients. The presence of an EML4-ALK translocation with concomitant EGFR/KRAS mutations is very rare among lung cancer patients. Our results indicate that the detection of the EML4-ALK translocation in subgroups of patients with NSCLC is crucial for applying targeted therapy.

## Materials and Methods

### Patients and samples

Samples were obtained from 208 NSCLC patients who underwent surgical resection of primary lung cancer at the Department of Lung Cancer Surgery, Tianjin Medical University General Hospital for diagnosis and treatment during 2006–2010. Written informed consent was obtained, and the study was approved by the Institutional Ethics Committee of Tianjin Medical University General Hospital. The inclusion criteria were: (1) surgical treatment without prior chemotherapy or treatment with EGFR-TKIs; (2) clear diagnosis of NSCLC; and (3) availability of tissues for biomarker studies. Clinical and pathological characteristics of the patients included are detailed in Table 1. Lung cancer staging for each patient was performed according to the AJCC Cancer Staging Manual, 7th edition. Patients in this study were disproportionately classified into stage III, because this stage of lung cancer is associated with surgical treatment. Survival time was calculated from the day of resection until April 6, 2011. Resected lung tissues were immediately immersed in liquid nitrogen.

H2228,human lung adenocarcinoma cell line with EML4-ALK fusion gene, was from the American Tissue Culture Collection (ATCC), and was maintained in DMEM containing 10% fetal bovine serum (GIBCO) at 37°C with 5% CO_2_.

### RNA isolation and reverse transcription

Frozen tissues (50–100 mg) were ground into a powder in liquid nitrogen and were suspended in 1 ml TRIzol reagent (Invitrogen, USA) for total RNA extraction, according to the manufacturer's protocol. Total RNA was quantified using a spectrophotometer (Beckman-Coulter, USA), and its quality was assessed by agarose gel electrophoresis. Total RNA (2 µg) was reverse-transcribed (RT) using M-MLV reverse transcriptase (Promega, USA), with minor modifications to the manufacturer's protocol. Briefly, the RNA template and random primers were incubated at 70°C for 10 min to melt the template secondary structure and were cooled on ice for 2 min. The complete reaction mixture was then incubated at 42°C for 60 min and at 70°C for 15 min.

### Nested RT-PCR detection of the EML4-ALK fusion gene

Nested RT-PCR was performed to amplify the EML4-ALK fusion gene. The first pair of primers was: forward 5′-ACCTAGAGAACGAGCGGGTCAG-3′, reverse 5′-TCGGCAAAGCGGTGTTGATTAC-3′. cDNA was amplified for 35 cycles at 94°C for 30 s, 60°C for 30 s, and 72°C for 3.5 min, followed by 7 min extension at 72°C. The first-run PCR product (2 µl) was then applied as the template for the second PCR. The second pair of primers was: forward 5′-CCACTCTGTCGGTCCGCTGAAT-3′, reverse 5′-CGGACACCTGGCCTTCATA CAC-3′. Samples were amplified for 30 cycles at 94°C for 30 s, 64°C for 30 s, and 72°C for 1 min, followed by 7 min extension at 72°C. Glyceraldehyde-3-phosphate dehydrogenase (GAPDH) was amplified as an internal control (5′-GTCAGTGGTGGACCTGACCT-3′ and 5′-TGAGCTTGACAAAGTGGTCG-3′). RT-PCR experiments for EML4-ALK positive cases were independently replicated three times, and amplified fragments were sequence-verified in both directions by the Beijing Genomics Institute (BGI, Beijing, China.).

### Immunohistochemistry and immunofluorescence

Serial 4-µm-thick sections from paraffin-embedded conventional tissues were deparaffinized in xylene and hydrated in a series of graded alcohols. Heat-induced antigen retrieval was carried out by microwave pretreatment in 5 mM Tris-HCl, pH 10.0, for 15 min. Mouse monoclonal anti-ALK antibody (DAKO, Carpinteria, CA) was used at a dilution of 1∶50 at 4°C overnight. Appropriate positive and negative controls were used for each experiment. Cytoplasmic staining was considered positive for ALK.

Immunofluorescence labeling was performed as previously described [26]. Anti-ALK antibody was used as described above. Rabbit polyclonal anti-Ki-67 antibody was purchased from ZSGB-Bio (ZSGB-Bio, Beijing, China), and was used at a dilution of 1∶200 at 4°C overnight. After washing 3 times with PBS, the slides were incubated with Flur488-conjugated (green, for ALK) and Alexa Fluor594-conjugated (red, for Ki-67) at 1∶100 dilution for 60 min at 37°C. The slides were counterstained with DAPI for 10 min at room temperature. Ki-67 was used as a control, which is strictly associated with cell proliferation and can be exclusively detected in the nucleus.

### EGFR and KRAS mutation analysis

Exons 18–21 in EGFR correspond to four tyrosine kinase domains that are frequently mutated in lung cancers. RT-PCR was used to identify these EGFR mutations in study samples. Thermal cycling consisted of an initial incubation at 95°C for 10 min, 35 cycles of 94°C for 30 s, 51°C for 30 s, and 72°C for 1 min, followed by 7 min extension at 72°C. The primers used were: forward 5′-GAGCTTGTGGAGCC TCTTAC-3′, reverse 5′-GCAGGGATTCCGTCATATGGCT-3′. KRAS gene mutations occur frequently in lung cancers and correspond to the effect of EGFR-TKI treatment in lung cancer patients. RT-PCR was also used for KRAS mutation analysis of exons 2 and 3, which includes most mutations of KRAS/codons 12, 13 and 16. cDNA was amplified for 35 cycles at 94°C for 30 s, 57°C for 30 s, and 72°C for 30 s, followed by 7 min extension at 72°C. The primers were: forward 5′-ATTTCGGACTGGGAGCGAGC-3′, reverse 5′-GCTGTGTCGAGAATAT CCAA-3′. All PCR products were sequence-verified in both directions.

### Analysis of clinicopathological parameters

Statistical analyses were performed using SPSS, v. 13.0 (Chicago, IL). Correlations between EML4-ALK mutations, clinical characteristics, and EGFR/KRAS status were evaluated using a nonparametric test. The overall survival of lung cancer patients was examined by Kaplan–Meier analysis. Statistical significance was set at p<0.05.

### Meta-analysis of clinical characteristics associated with EML4-ALK translocation

The English-language medical literature was reviewed using the PubMed database (National Center for Biotechnology Information, NCBI) with the search term “EML4-ALK.” The reports selected involved clinical research on EML4-ALK translocation frequency in human NSCLC tumor tissues. Studies that were *in vitro* in nature, involved non-lung cancer tissues, assessed EML4-ALK amplification, or lacked pathological data were excluded. A total of 28 articles were returned in the initial search and only 14 were left after excluding irrelevant studies. The number of patients in each study, the number of patients with EML4-ALK translocation, the proportion of each variant of EML4-ALK translocation, and the patients' pathology type, age, gender, and smoking status were extracted from each publication (Table 4). Each report was organized into tables according to the following questions: (1) What is the range of EML4-ALK translocation frequencies in NSCLC? (2) Is pathology type, gender, smoking status, or age significantly associated with EML4-ALK translocation? The odds ratio (OR) and 95% CI were calculated using Review Manager 4.2 software (the Cochrance Co. Oxford, UK). In populations that were not significantly heterogeneous, the Mantel–Haenszel fixed effects model was applied to the data, and the OR and 95% CI were determined. For significantly heterogeneous populations, the DerSimonian–Laird random effects model was used in a sensitivity analysis. Funnel plots were made using SAS 9.0 software (SAS Institute INC, NC), and Egger's test was applied to evaluate article bias.

## Supporting Information

Table S1 **Clinical features of patients with EGFR exons mutations.**
(DOC)
