# Supplementary material for: Clinical Significance of EML4-ALK Fusion Gene and Association with EGFR and KRAS Gene Mutations in 208 Chinese Patients with Non-Small Cell Lung Cancer
Source: PLoS One. 2013 Jan 14;8(1):e52093. doi: 10.1371/journal.pone.0052093 (PMC3544857; doi:10.1371/journal.pone.0052093)
Supplement: Table S1 — Clinical features of patients with EGFR exons mutations. (DOC) [file pone.0052093.s001.doc]

**Supporting information**

**Table S1. Clinical features of patients with EGFR exons mutations**

| **Case number** | **Age** | **Gender** | **Smoking status** | **Pathology**  **（H&E）** | **P-TNM** | **Metastasis status** | **EGFR exons**  **abnormal** | **EML4-ALK** | **KRAS**  **mutation** |
| --- | --- | --- | --- | --- | --- | --- | --- | --- | --- |
| 6 | 75 | M | Y | Ad | T2aN2M0 | M | E746－A750del | (-) | (-) |
| 20 | 51 | F | N | SCC | T2bN2M0 | M | L858R | (-) | (-) |
| 28 | 60 | M | Y | Ad | T2aN2M0 | M | P843L，L858R | (-) | (-) |
| 30 | 65 | F | N | Ad | T2aN2M0 | M | L858R | (-) | (-) |
| 32 | 62 | F | N | Ad | T2N1M0 | M | L858R | (-) | (-) |
| 36 | 58 | M | N | Ad | T3N0M0 | N | L858R | (-) | (-) |
| 76 | 73 | M | Y | Ad | T2bN2M0 | M | L858R | (-) | (-) |
| 84 | 51 | F | N | Ad | T2aN0M1 | M | L858R | (-) | (-) |
| 104 | 42 | M | N | Ad | T2aN3M0 | M | E746－A750del | (-) | (-) |
| 118 | 72 | F | N | Ad | T2aN0M0 | N | L858R | (-) | (-) |
| 132 | 45 | M | N | Other | T3N3M0 | M | L858R | (-) | (-) |
| 134 | 65 | M | N | SCC | T2aN2M0 | M | E746－A750del | (-) | (-) |
| 136 | 71 | M | N | SCC | T2aN1M0 | M | L861Q,G719A | (-) | (-) |
| 156 | 40 | F | N | SCC | T4N2M0 | M | G724S,P769L | (-) | (-) |
| 164 | 55 | F | Y | Ad | T2aN1M0 | M | L858R | (-) | (-) |
| 166 | 56 | F | N | Ad | T2N2M0 | M | L858R | (-) | (-) |
| 178 | 58 | M | Y | Ad | T2N0M0 | N | E746－A750del | (-) | (-) |
| 180 | 69 | F | N | Ad | T2aN0M0 | N | E746－A750del | (-) | (-) |
| 186 | 53 | M | Y | Ad | T2N0M0 | N | E746－A750del | (-) | (-) |
| 200 | 78 | F | Y | Ad | T1N3M0 | M | L858R | (-) | (-) |
| 204 | 59 | M | Y | Ad | T3N1M0 | M | E746－A750del | (-) | (-) |
| 206 | 41 | M | N | Ad | T2aN2M1 | M | E746－A750del | (-) | (-) |
| 220 | 73 | M | N | Ad | T2aN2M1 | M | L858R,S768I | (-) | (-) |
| 226 | 64 | F | N | Ad | T2aN0M0 | N | E746－A750del | (-) | (-) |
| 250 | 66 | M | Y | Ad | T4N2M0 | M | L858R | (-) | (-) |
| 254 | 68 | F | N | Ad | T2aN1M0 | M | L858R | (-) | (-) |
| 260 | 46 | F | N | SCC | T2aN3M0 | M | E746－A750del | (-) | (-) |
| 262 | 67 | F | N | Ad | T2aN1M0 | M | E746-A750del | (-) | (-) |
| 266 | 67 | F | Y | Ad | T3N1M0 | M | E746－A750del | (-) | (-) |
| 274 | 52 | F | N | Ad | T3N2M0 | M | E746－A750del | (-) | (-) |
| 276 | 67 | F | N | Ad | T2aN0M0 | N | E746－A750del | (-) | (-) |
| 284 | 61 | F | N | Ad | T2aN2M1 | M | L858R | (-) | (-) |
| 288 | 61 | F | N | Ad | T2bN0M0 | N | L858R | (-) | (-) |
| 312 | 64 | M | Y | Ad | T2N2M0 | M | L833P，H835L（CA/TC） | (-) | (-) |
| 316 | 54 | M | Y | Ad | T2aN0M0 | N | G719S | (-) | (-) |
| 320 | 72 | F | Y | Ad | T4N0M0 | N | L858R | (-) | (-) |
| 328 | 61 | F | N | Ad | T2aN0M0 | N | E746－A750del | (-) | (-) |
| 334 | 46 | F | N | Ad+SCC | T3N0M0 | N | L858R | (-) | (-) |
| 336 | 60 | F | N | Ad | T2aN2M0 | M | L858R | (-) | (-) |
| 344 | 58 | F | N | Ad | T2aN3M0 | M | L858R | (-) | (-) |
| 350 | 60 | M | Y | Ad | T4N3M0 | M | L858R | (-) | (-) |
| 358 | 44 | F | N | Ad | T1N1M0 | M | E746－A750del | (-) | (-) |
| 360 | 47 | M | Y | Ad+SCC | T4N1M0 | M | E746－A750del | (-) | (-) |
| 370 | 77 | F | N | Ad | T2aN0M0 | N | E746－A750del | (-) | (-) |
| 376 | 33 | F | N | Ad | T2aN2M0 | M | E746－A750del | (-) | (-) |
| 386 | 47 | F | N | Ad | T3N2M1 | M | E746-A750del | (-) | (-) |
| 390 | 71 | M | Y | Ad+SCC | T2N2M0 | M | L858R | (-) | (-) |
| 408 | 68 | F | Y | Ad | T2aN0M0 | N | L747-E749del；A750P | (-) | (-) |
| 412 | 64 | M | N | Ad | T2N2M1 | M | A767-P769dupASP | (-) | (-) |
| 414 | 57 | M | Y | Ad | T2aN3M0 | M | L858R | (-) | (-) |
| 418 | 48 | M | Y | Ad | T2bN2M0 | M | P772-H773dupPH | (-) | (-) |
